#  Real-Time Imaging of Incision-Related Descemet Membrane Detachment During Cataract Surgery

**DOI:** 10.1001/jamaophthalmol.2020.5396

**Published:** 2020-12-10

**Authors:** Ye Dai, Zhenzhen Liu, Wei Wang, Bo Qu, Jianping Liu, Nathan Congdon, Mingguang He, Lixia Luo, Yizhi Liu

**Affiliations:** 1 State Key Laboratory of Ophthalmology, Zhongshan Ophthalmic Center, Sun Yat-sen University, Guangzhou, Guangdong, China; 2 Translational Research for Equitable Eye Care, Centre for Public Health, Royal Victoria Hospital, Queen’s University Belfast, Belfast, United Kingdom

## Abstract

**Importance:**

Incision-related Descemet membrane detachment (DMD) is a common complication of cataract surgery. Most postoperative severe DMD that leads to corneal decompensation originates from intraoperative incision-related DMD. It is important to determine the incidence, extent, and associated risk factors of intraoperative DMD at each step of surgery to help in formulating precise and effective prevention strategies.

**Objectives:**

To investigate the intraoperative development of incision-site DMD associated with a 2.2-mm clear corneal incision during cataract surgery and to analyze its associated factors.

**Design, Setting, and Participants:**

In this case series, consecutive, prospectively enrolled 133 patients with cataract 50 to 90 years of age (133 eyes) undergoing coaxial 2.2-mm clear corneal microincision phacoemulsification with intraocular lens (IOL) implantation between January 1 and March 31, 2019, at Zhongshan Ophthalmic Center, Sun Yat-sen University, Guangzhou, China, were studied.

**Exposures:**

Coaxial 2.2-mm clear corneal microincision phacoemulsification with IOL implantation.

**Main Outcomes and Measures:**

Real-time incidence and extent of intraoperative incision-related DMD at each step of surgery.

**Results:**

Among 133 patients with cataracts (mean [SD] age, 72.3 [8.1] years; 77 [57.9%] female), DMD was encountered in 125 eyes (94.0%), occurring at the following steps: capsulorrhexis (2 [1.6%]), hydrodissection (7 [5.6%]), phacoemulsification (69 [55.2%]), irrigation-aspiration (44 [35.2%]), and IOL implantation (3 [2.4%]). The extent of DMD increased during the operation (mean [SD] difference between final and initial relative DMD length, 22.8% [1.4%]; 95% CI, 20.0-25.6; *P* < .001). Associations for the extent of DMD found in multivariate stepwise analyses included time of ultrasonography (β = 0.34; 95% CI, 0.17-0.50; *P* < .001), equivalent mean ultrasonic power (β = 87.8; 95% CI, 19.1-156.4; *P* = .01), and the presence of DMD at the anterior and posterior wound margins (coefficient = 16.7; 95% CI, 6.4-26.9; *P* = .002).

**Conclusions and Relevance:**

The results of this case series suggest that friction of surgical instruments has the greatest association with incisional DMD. Decreasing ultrasonic energy and phacoemulsification time may reduce the severity of incisional DMD.

## Key Points

**Question:**

When and how does incision-related Descemet membrane detachment (DMD) occur during cataract surgery?

**Findings:**

In this case series of 133 patients with cataract, DMD was found in 125 cataract operations (94.0%) and occurred mostly during the phacoemulsification step (69 cases [55.2%]); DMD also increased throughout surgery.

**Meaning:**

These findings suggest that incision-related DMD mainly occurs during the surgical steps in which the instruments create the greatest friction at the incision site and that severity is associated with the level of ultrasonic energy and length of time of phacoemulsification.

## Teaser

This case series of patients with cataract investigates the intraoperative development of incision-site Descemet membrane detachment associated with a 2.2-mm clear corneal incision during cataract surgery and its associated factors.

## Introduction

Incision-related Descemet membrane detachment (DMD) is a common complication in cataract surgery. Previous studies^1,2,3^ have found that the incidence of DMD at the incision site 1 day after phacoemulsification is high, ranging from 36.7% to as high as 82.0%. Slight incisional DMD can be self-healing. However, in the case of inexperienced surgeons or unhealthy corneas, severe DMD may occur, leading to corneal decompensation that requires transplantation.^4,5,6,7^ The surgical steps during which incisional DMD is most likely to be initiated remain unknown, as do the potential factors that contribute to or reduce the risk of DMD, which limits the ability of surgeons to formulate effective prevention strategies. We used intraoperative optical coherence tomography (iOCT) technology to detect the occurrence of incisional DMD in real time during each step of phacoemulsification and analyzed associated factors to provide an evidence base for specific prevention strategies.

## Methods

### Participants

In this case series, consecutive patients 50 to 90 years of age undergoing phacoemulsification with intraocular lens (IOL) implantation for age-related cataract at Zhongshan Ophthalmic Center between January 1 and March 31, 2019, were prospectively enrolled. Only right eyes were included for patients undergoing surgery in both eyes. Exclusion criteria included the presence in the operative eye of corneal abnormalities (eg, Fuchs corneal dystrophy), glaucoma, uveitis, inability of the pupil to dilate to 6 mm or more, previous ocular surgery, known or suspected posterior capsular rupture, lens dislocation, or Lens Opacities Classification System III nuclear opalescence grading score greater than 6.0.^8^ This study was approved in advance by the ethics committee at the Zhongshan Ophthalmic Center, Sun Yat-sen University, Guangzhou, China. Written informed consent was obtained from all participants, and all data were deidentified. The study was conducted in accordance with the principles of the Declaration of Helsinki.^9^ Patients were not offered any compensation or incentives to join this study. The study followed the reporting guideline for case series.

### Operative Procedures

All operations were performed by an experienced ophthalmologist (Y.L.) following a standardized procedure. One drop each of 0.5% topical tropicamide (Shenyang Xingqi) and 0.5% promecaine hydrochloride (Novartis) were administered to the surgical eye every 5 minutes a total of 3 times before surgery. A temporal, 2-plane clear corneal incision was created with a 2.2-mm keratome (Alcon Labs). Injection of an ophthalmic viscoelastic device that consisted of medical sodium hyaluronate gel (Hangzhou Singclean Medical Products Co Ltd) was used to maintain the stability of the anterior chamber. A 26-gauge capsulotomy needle was used to create a continuous circular capsulorrhexis of 5.5 to 6.0 mm in diameter. Hydrodissection was performed through the main incision. A Centurion Vision System (Alcon Labs) device was used to perform phacoemulsification surgery, including nucleus chopping with a 0.9-mm U/S tip (Centurion OZil handpiece; Alcon Labs) and a straight-headed coaxial tip for irrigation-aspiration. The aspiration flow rate was set as 35 mL/min, and the vacuum level was set as 500 mm Hg in linear mode during irrigation-aspiration. Torsional phacoemulsification was set between 60% and 100%, suction velocity was 33 to 35 mL/min, and negative pressure was maintained in the range of 330 to 350 mm Hg during phacoemulsification. A single-focus IOL (Alcon Labs) was implanted. The surgeon created the incision and then placed the phacoemulsification tip, irrigation-aspiration tip, and other instruments through the incision without any forceps facilitation. Intraoperative parameters, including surgical time, cumulative dissipated energy (CDE), ultrasonography time (UST), and equivalent mean ultrasonic energy (displayed as footswitch position 3 [FP3]), were recorded. The CDE was defined as UST × FP3.^10,11,12^

### Intraoperative Observation and Recording

The operating microscope (Opmi Lumera 700; Carl Zeiss Meditec) parameters were as follows: 65% light intensity and magnification of ×7.5. An iOCT system (Zeiss Rescan 700; Carl Zeiss Meditec) was connected to the operating microscope to obtain real-time, intraoperative scanning results at a scanning mode of 5 lines, a spacing of 0.75 mm, and a size of 6 mm. Sectional images at each of the 5 scanning lines were simultaneously obtained in 1 scan at a scanning depth of approximately 2.0 mm. The 5 scanning lines were adjusted during each procedure so that they were evenly distributed and perpendicular to the incision. A screenshot was taken and saved when the sectional view of the longest DMD throughout the procedure was located at the third (central) scan line (eFigure 1 in the Supplement). The positions of DMD that occurred at the incision were classified as anterior lip (eFigure 2 in the Supplement), posterior lip (eFigure 2 in the Supplement), or both.

### Measurement and Comparison of the Extent of Intraoperative DMD

Images and videos of intraoperative DMD obtained by iOCT were collected and compared using Photoshop CS5 (Adobe Systems Software Ltd). The central scan line images exported from the iOCT were all 454 × 308 pixels (160.16 × 108.66 mm at a resolution of 96 dpi). All images were measured and compared by the same observer (Y.D.) using the ruler function of Photoshop (Adobe) (eFigure 2 in the Supplement). The initial length of DMD was defined as the DMD length when first detected intraoperatively using iOCT. The final length of DMD was defined as the DMD length at the end of surgery. The longest detachment length among all participants was taken as 100%, with the relative detachment length of other participants defined as percentages relative to this value. The DMD length of participants with simultaneous anterior and posterior lip detachment was defined as the sum of the DMD length of anterior and posterior lip.

### Statistical Analysis

The Kolmogorov-Smirnov test was used to confirm normality of the distribution of continuous variables. The paired *t* test was used to compare the initial and final extent of intraoperative DMD for participants. Univariate, age- and sex-adjusted, and multivariate linear regression models were used to explore potential associations with the extent of intraoperative DMD. Variables with *P* < .10 were entered into a stepwise multivariate analysis using the forward method. All statistical analyses were performed using Stata MP software, version 14.0 (StataCorp). *P* values were 1- or 2-sided, and no adjustments to *P* values were made for the multiple analyses undertaken.

## Results

**Figure 1.**
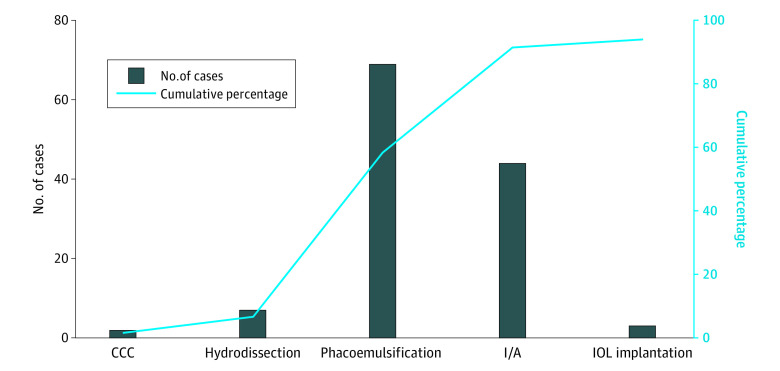
Dynamic Profile of Descemet Membrane Detachment Occurrence During Cataract Surgery For each patient, intraoperative scanning images were captured during 5 surgical steps, including capsulorrhexis (CCC), hydrodissection, phacoemulsification, irrigation and aspiration (I/A), and intraocular lens (IOL) implantation.

**Figure 2.**
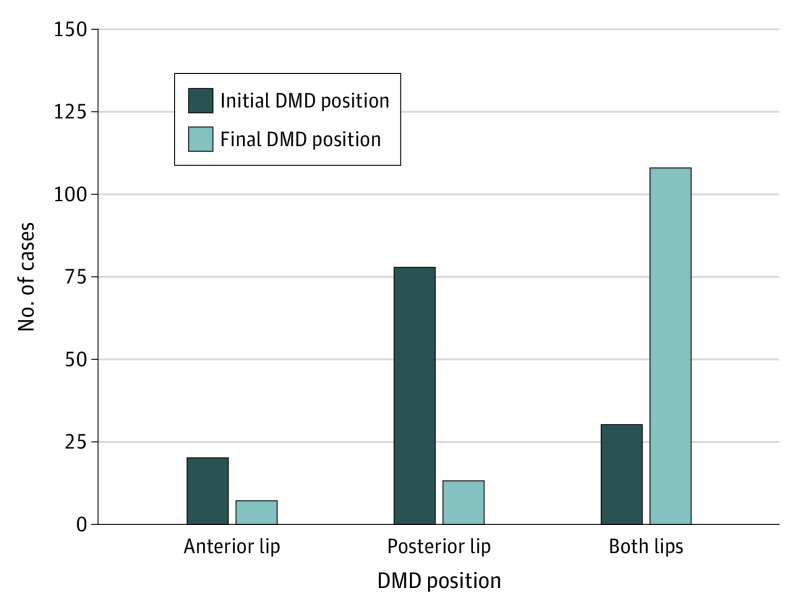
Comparison of Initial Descemet Membrane Detachment (DMD) Position with Final DMD Position

Among 133 patients with cataract (mean [SD] age, 72.3 [8.1] years; 77 [57.9%] female) (Table 1), DMD associated with the 2.2-mm microincision was observed intraoperatively in 125 (94.0%) by iOCT. Among these patients, 2 (1.6%) experienced DMD during capsulorrhexis, 7 (5.6%) during hydrodissection, 69 (55.2%) during phacoemulsification, 44 (35.2%) during irrigation-aspiration, and 3 (2.4%) during IOL implantation (Figure 1). The initial DMD occurred most frequently at the posterior margin of the surgical wound (n = 77 [57.9%]). At the end of surgery, the final DMD occurred at the anterior wound margin (n = 6 [4.5%]), posterior wound margin (n = 12 [9.0%]), and both margins (n = 107 [80.4%]) (Figure 2). The length of the DMD extended during surgery (mean [SD] difference between final and initial relative DMD length, 22.8% [1.41%]; 95% CI, 20.0%-25.6%; *P* < .001). The univariate regression model of potential factors associated with DMD found that the extent of incision-site DMD was positively correlated with older age (β = 0.57; 95% CI, 0.05-1.09; *P* = .03), greater nuclear hardness (β = 5.94; 95% CI, 1.55-10.33; *P* = .008), presence of DMD at both margins of the incision (β = 17.69; 95% CI, 4.43-30.94; *P* = .009), CDE (β = 2.23; 95% CI, 1.41-3.06; *P* < .001), UST (β = 0.36; 95% CI, 0.19-0.53; *P* < .001), and FP3 (β = 99.64; 95% CI, 23.90-175.30; *P* = .01) (Table 2). In the multivariate regression model, DMD at both wound margins (β = 16.68; 95% CI, 6.43-26.93; *P* = .002), UST (β = 0.34; 95% CI, 0.17-0.50; *P* < .001), and FP3 (β = 87.77; 95% CI, 19.11-156.42; *P* = .01) were independently associated with greater extent of DMD (Table 2).

**Table 1.** Baseline Characteristics of the Study Patients

Characteristic	Finding^a^
Age, mean (SD), y	72.3 (8.1)
Sex	
Male	56 (42.1)
Female	77 (57.9)
LOCS III grade, mean (SD)	3.80 (0.91)
Endothelial cell density, mean (SD), mm^2^	2630 (320)
Hypertension present	57 (42.9)
Diabetes present	27 (20.3)

Abbreviation: LOCS III, Lens Opacities Classification System III.

^a^ Data are presented as number (percentage) of patients unless otherwise indicated.

**Table 2.** Analysis of Risk Factors for the Extent of Intraoperative Incision-Site DMD

Factor	Univariate analysis		Stepwise multiple logistic regression		
	β (95% CI)	*P* value		β (95% CI)	*P* value
Age	0.57 (0.05 to 1.09)	.03		NA	NA
Sex	–3.22 (–11.40 to 4.97)	.44		NA	NA
Presence of hypertension	–3.15 (–13.30 to –7.00)	.54		NA	NA
Presence of diabetes	–3.15 (–13.30 to –7.00)	.54		NA	NA
Total surgery time	0.01 (–0.08 to –0.10)	.79		NA	NA
UST	0.36 (0.19 to 0.53)	<.001		0.34 (0.17 to 0.50)	<.001
CDE	2.23 (1.41 to 3.06)	<.001		NA	NA
FP3	99.64 (23.90 to 175.30)	.01		87.77 (19.11 to 156.42)	.01
ECD	–0.007 (–0.02 to 0.006)	.30		NA	NA
Nuclear hardness	5.94 (1.55 to 10.33)	.008		NA	NA
DMD at both lips	17.69 (4.43 to 30.94)	.009		16.68 (6.43 to 26.93)	.002

Abbreviations: CDE, cumulative dissipated energy; DMD, Descemet membrane detachment; ECD, endothelial cell density; FP3, footswitch position 3 (equivalent mean ultrasonic power); NA, not applicable; UST, ultrasonography time.

## Discussion

This case series is, to our knowledge, the first study to use real-time iOCT to assess dynamic changes in incision-site DMD during 2.2-mm microincisional phacoemulsification. This study found the incidence of intraoperative incision-site DMD to be 94%, far higher than reports based on postoperative examination, in which rates have ranged from 36.7% to 82%.^1,2,3^ This difference may be attributable to much more incisional DMD ignored by static observation from conventional anterior OCT for lack of iOCT, which can dynamically observe the incision site in real time. The structure and size of the incision could affect the incidence of DMD. A previous study^2^ found that the incidence of DMD in a 2.2-mm incision group was significantly higher than in a 2.85-mm group at postoperative day 1, which indicated that the smaller the incision, the higher the incidence of DMD.

Incision-site DMD occurred as early as the capsulorrhexis phase and was observed most commonly during the phacoemulsification and irrigation-aspiration steps. Descemet membrane detachment was initially most commonly observed at the posterior margin of the corneal incision and increased in length during surgery. These results suggest that the occurrence and severity of DMD are likely related to intraoperative manipulation of surgical instruments. Steps such as nuclear chopping, phacoemulsification, and aspiration all use the corneal incision, especially the posterior margin, as a fulcrum. The diameter of the surgical instrument exactly matches the incision size and is larger than that of the syringe used for capsulorrhexis and hydrodissection. An animal study by Vasavada et al^13^ found that regular vibration of the phacoemulsification tip and mechanical activity can cause incision-site DMD during phacoemulsification. Previous studies^14,15,16^ have reported corneal incision enlargement during surgery, more prominently with smaller incisions, including a total wound enlargement of 11.4% with a 1.8-mm incision. This finding suggests that intraoperative manipulation of instruments may cause expansion of the incision. As opposed to well-constructed incisions created with a keratome, the irregularity of the enlarged incision may affect wound healing, especially at the internal aspect.^17^ Moreover, the friction of repeated entry of instruments into the anterior chamber through the incision is a known culprit in the extension of DMD,^18,19^ and reducing this friction with an enlarged and open wound may reduce DMD. Therefore, we have conducted another study that modified the architecture of incision for reducing the incidence of DMD (Y. Dai, MD, PhD, unpublished data, 2019-2020).

The univariate analyses found that DMD at both margins of incision, age, nuclear hardness, UST, FP3, and CDE were positively associated with the extent of DMD. Previous studies^20,21,22,23^ have found that greater nuclear hardness may require longer UST, higher FP3, and greater CDE. This finding suggests the likelihood of collinearity between nuclear hardness and the above-mentioned surgical parameters and may explain why nuclear hardness was weakened in the multivariate regression model. In the present study, the operations were performed by the same experienced surgeon (Y.L.) for the consistency of interventions, with the aspiration flow rate set at 35 mL/min and the vacuum level set at 500 mm Hg in linear mode during irrigation-aspiration. It is worth exploring whether the aspiration flow rate and vacuum level influence the incidence and aggravation of DMD in the future.

On the basis of this study, several strategies may be of use to reduce intraoperative DMD. For example, handpiece types and materials could be optimized to prevent DMD caused by mechanical friction, or the construction of the incision could be modified for the enlarged incision mechanically at the end of surgery.^14,18^ A previous study^19^ found that femtosecond-laser clear corneal incisions have less incision-site DMD compared with keratome-assisted clear corneal incisions. Furthermore, using a femtosecond laser to complete nucleus fragmentation could reduce the required amount of operative UST and energy needed.^24^ Enlarging the internal size of the incision with the femtosecond laser could increase the scope for movement of the phacoemulsification handpiece and irrigation-aspiration tip without compromising the stability of the incision or increasing surgery-induced astigmatism in the future.

### Limitations

The results of this study should be assessed within the context of its limitations. The study included only age-related cataracts to focus on the impact of common surgical procedures on DMD; therefore, the results are not necessarily applicable to patients undergoing cataract extractions for other reasons. Patients with corneal lesions were excluded as well, which may underestimate the possible impact of incision-related DMD on affected patients. The architecture of the corneal incision is known to affect its stability,^25^ but only one such approach was evaluated in the present study. To evaluate the profile and prognosis of intraoperative incision-related DMD more comprehensively, patients with a wider range of cataract causes (eg, traumatic, metabolic, and inflammatory), corneal pathologies (especially lower corneal endothelial cell density and corneal endothelial defects), and corneal incisions of different architecture should be studied. Moreover, this study focused on the occurrences and extent of intraoperative DMD, which no studies observed during cataract surgery for lack of iOCT, and the evaluation of postoperative outcomes was therefore not included. Whether the extent of DMD would be associated with changes in corneal endothelial cell counts after cataract surgery is worth exploring in the future. In addition, the wide CIs reflective of the relatively small sample size in this study precluded complete understanding of the magnitude of the results.

## Conclusions

These results suggest that friction of surgical instruments may have the greatest association with incisional DMD. Decreasing FP3 and phacoemulsification time may be associated with reductions in the severity of incisional DMD.

## Notes

Supplement. **eFigure 1.** Screenshot of the Intraoperative Optical Coherence Tomography (iOCT) Display Interface **eFigure 2.** Measurement of the Length of DMD
