# Supplementary material for: Real-Time Imaging of Incision-Related Descemet Membrane Detachment During Cataract Surgery
Source: JAMA Ophthalmol. 2020 Dec 10;139(2):150–5. doi: 10.1001/jamaophthalmol.2020.5396 (PMC7729572; doi:10.1001/jamaophthalmol.2020.5396)
Supplement: Supplement. — eFigure 1. Screenshot of the Intraoperative Optical Coherence Tomography (iOCT) Display Interface eFigure 2. Measurement of the Length of DMD [file jamaophthalmol-e205396-s001.pdf]

Supplemental Online Content

Dai Y, Liu , Wang W, et al. Real-time imaging of incision-related Descemet membrane detachment during cataract surgery. *JAMA Ophthalmol.* Published online December 10, 2020. doi:10.1001/jamaophthalmol.2020.5396

eFigure 1. Screenshot of the Intraoperative Optical Coherence Tomography (iOCT) Display Interface

eFigure 2. Measurement of the Length of DMD

This supplemental material has been provided by the authors to give readers additional information about their work.

© 2020 Dai Y et al. *JAMA Ophthalmology.*

eFigure 1. Screenshot of the intraoperative optical coherence tomography (iOCT) display interface. A. Intraoperative image of the surgical field. Five scanning lines were placed perpendicularly to the incision. **B-F.** Real-time sectional view of the corneal incision. Descemet membrane's detachment (DMD) on the post lip of the incision is shown in panel D (red arrow).

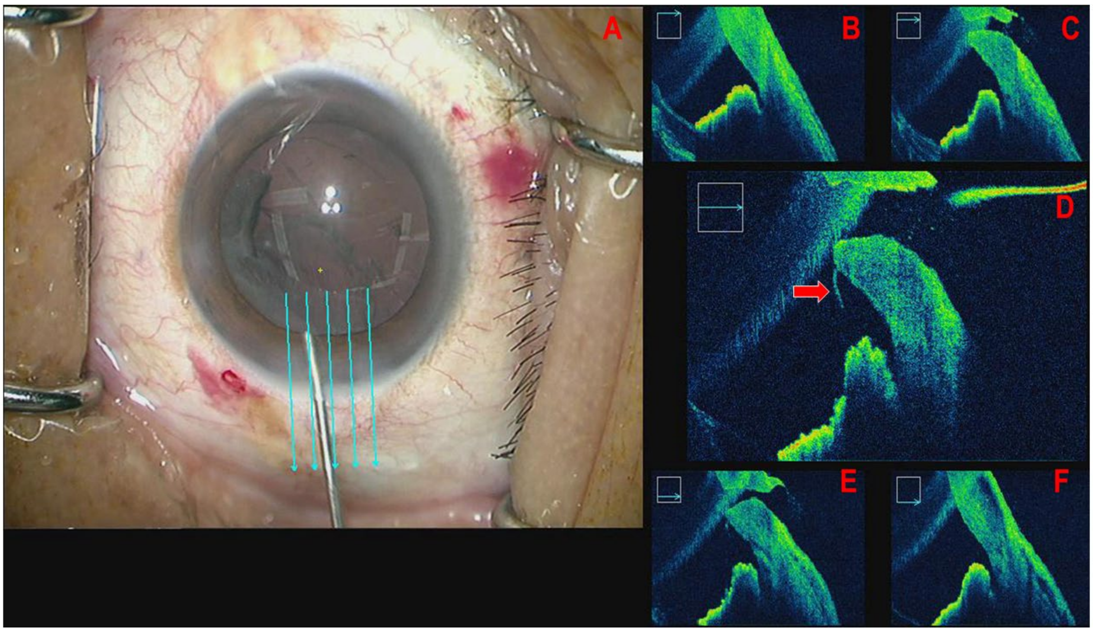

© 2020 Dai Y et al. *JAMA Ophthalmology.*

eFigure 2. Measurement of the length of DMD. All intraoperative images were captured with the same magnification and with a resolution of 96dpi. The length of DMD was measured in pixels with the ruler tool of Photoshop CS5 (red rectangle). The location of incision-site DMD in this study was classified as anterior lip (red arrow) and posterior lip (yellow arrow). This iOCT image showed the DMD occurred at the anterior lip of incision (red arrow).

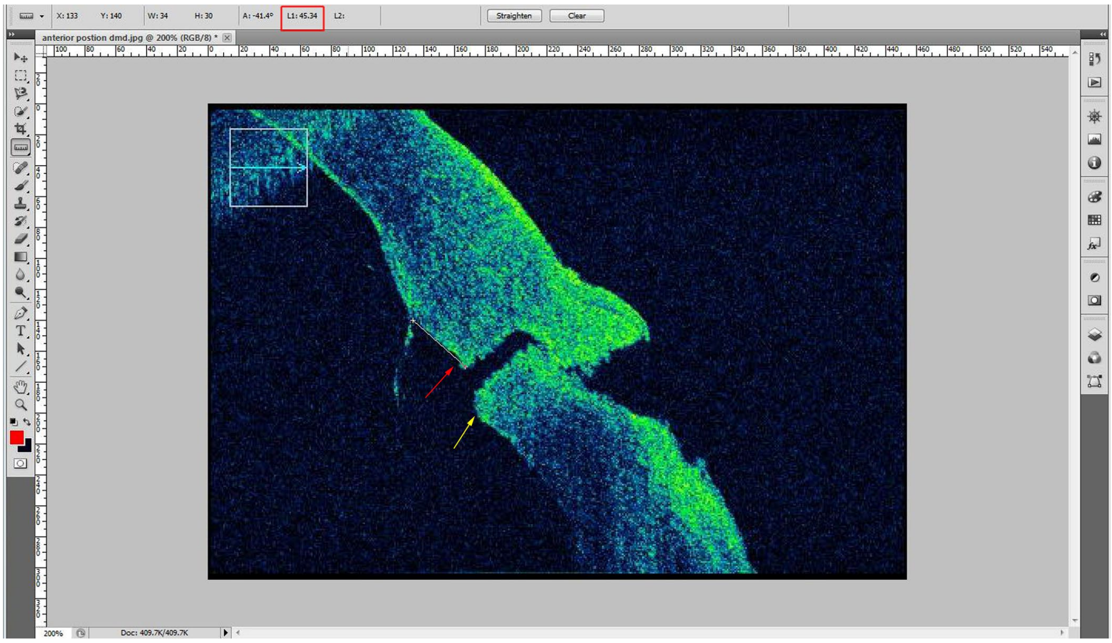

© 2020 Dai Y et al. *JAMA Ophthalmology.*
